# Association of Arterial Hypertension with Thoracic Spondylophyte Formation: A Secondary Analysis of Cross-Sectional MRI Data from the SHIP Cohort

**DOI:** 10.3390/healthcare14081024

**Published:** 2026-04-13

**Authors:** Kim Lisa Westphal, Fiona Mankertz, Lukas Rasche, Robin Bülow, Mark Oliver Wielpütz, Marie-Luise Kromrey, Carolin Malsch

**Affiliations:** 1 Institute for Diagnostic Radiology and Neuroradiology, University Medicine Greifswald, 17475 Greifswald, Germany; kimlisawestphal@gmx.de (K.L.W.); robin.buelow@med.uni-greifswald.de (R.B.); mark.wielpuetz@med.uni-greifswald.de (M.O.W.); 2 Department for Radiology and Interventional Radiology, University Hospital Tübingen, 72076 Tübingen, Germany; fiona.mankertz@med.uni-tuebingen.de; 3 Institute for Mathematics and Computer Science, University Greifswald, 17489 Greifswald, Germany; lukas.rasche.2002@icloud.com (L.R.);; 4 Institute and Polyclinic for Diagnostic and Interventional Radiology, Faculty of Medicine, University Hospital Carl Gustav Carus Dresden, Technische Universität Dresden (TU Dresden), 01307 Dresden, Germany

**Keywords:** magnetic resonance imaging, vertebral body, hypertension

## Abstract

**Objective:** Back pain is a multifactorial condition commonly associated with degenerative spinal changes. Spondylophytes are frequent outgrowths of the vertebral bodies that may be influenced by arterial hypertension via a possible increased pulsation of the aorta and its effects on bone remodeling. If it can be demonstrated that an increased pulse pressure in the aorta due to hypertension promotes the growth of spondylophytes and thereby increases the likelihood of back pain, future studies may investigate how the effectiveness of blood pressure management can be improved in order to reduce the prevalence of degenerative changes in the spine and, consequently, prevent back pain. This study investigated the association between arterial hypertension and thoracic spondylophyte formation using whole-body MRI data from the population-based Study of Health in Pomerania (SHIP). **Materials and Methods:** Spondylophyte presence and area were assessed for their association with hypertension status in 859 SHIP-START-3 participants who underwent whole-body MRI. Right-sided spondylophytes at T8-T11 were measured on axial T2-weighted sequences. Hypertension was defined by self-report or antihypertensive medication use; a sensitivity analysis was conducted using the 2024 European Society of Cardiology definition (systolic blood pressure ≥ 140 mmHg). Multivariate regression models adjusted for age, sex, obesity, and smoking were used to assess associations. Machine learning algorithms were applied for validation. **Results:** Spondylophytes were present in 87.7% of participants. Hypertension was significantly associated with spondylophyte presence (OR = 2.07, 95% CI: 1.15–3.81) but not consistently associated with spondylophyte size. Spondylophyte size increased from T8 to T11, and was associated with age, male sex, and obesity. Sensitivity analyses widely confirmed robustness of the analysis. **Conclusions:** This population-based MRI study investigates the still insufficiently studied relationship between arterial hypertension and the formation of thoracic spondylophytes. The findings are consistent with the hypothesis that hypertension may be associated with spinal bone remodelling, though causal inference remains limited by the cross-sectional study design. Further longitudinal studies are needed to clarify causality and clinical relevance for spinal degeneration and back pain.

## 1. Introduction

Back pain remains a highly prevalent and economically significant health concern with a multifactorial aetiology, affecting more than two-thirds of the adult population in Germany each year and contributing substantially to healthcare utilization and economic burden [1,2]. Degenerative spinal changes are among the most frequent structural contributors, particularly in older adults, and include features such as intervertebral disc degeneration and spondylophyte formation [3]. Spondylophytes are osseous projections that arise at the margins of vertebral bodies and are commonly interpreted as a structural adaptation to chronic mechanical loading, with the aim of stabilizing the spinal column under stress [3,4]. There is evidence that spondylophytes, as degenerative changes in the spine, can be associated with back pain [5,6].

Anatomical observations suggest that regional vascular anatomy may influence spondylophyte development [7,8,9,10]. In particular, the anterior longitudinal ligament in the thoracic spine frequently shows asymmetric ossification, with comparatively reduced bone formation on the left side of the descending thoracic aorta. This pattern has been observed in patients with diffuse idiopathic skeletal hyperostosis and may be attributed to the pulsatile force of the aorta acting as an inhibitory mechanical stimulus. These findings support the hypothesis that local vascular dynamics can modulate bone remodelling along the spine [11,12,13].

Established risk factors for spondylophyte formation include increasing age [7,14,15], obesity [14,16], and mechanical strain [16,17]. However, the potential contribution of arterial hypertension has not been systematically studied. Given the association between elevated blood pressure and increased aortic wall stress, hypertension may modify mechanical stress to vertebral bodies through altered pulsatility, leading to microfissuration and increased bone growth at the bone–ligament interface. Beyond mechanical strain, hypertension is known to contribute to systemic vascular changes, including reduced microcirculation, endothelial dysfunction, and increased arterial stiffness. These processes may impair nutrient supply to spinal structures—particularly the vertebral endplates and intervertebral discs—thereby promoting degenerative changes. In addition, hypertension is associated with chronic low-grade inflammation and altered bone metabolism, both of which may contribute to osteophyte (spondylophyte) formation [18,19].

Despite these plausible mechanisms, there is limited evidence in large cohorts on whether hypertension contributes to spondylophyte development, although it has been shown that aortic pulsation has an inhibitory effect on spondylophyte formation on the left side of the spine at the level of T8 to T11, and that spondylophytes are more frequently formed on the right side of the vertebral bodies in this area. Previous studies have demonstrated that aortic pulsation exerts a localized inhibitory effect on spondylophyte formation on the left side of the thoracic spine at levels T8–T11, resulting in a right-sided predominance of ossification in this region [20]. However, these studies focused on the mechanical effect of direct aortic contact on lateralized bone formation, rather than on the systemic contribution of arterial hypertension to overall spondylophyte development. Whether elevated blood pressure, through increased aortic wall stress and altered pulsatility, contributes to spondylophyte formation independently of this localized mechanical effect has not been systematically investigated in large population-based cohorts. The present study therefore addresses this gap by examining the association between arterial hypertension and thoracic spondylophyte presence and size in a population-based MRI cohort.

The aim of this study was to investigate the association between arterial hypertension and thoracic spondylophyte formation using whole-body MRI data from the population-based SHIP cohort. Spondylophyte presence and area at vertebral levels T8 to T11 were analysed in relation to hypertension status considering relevant covariates selected based on a causal graph.

## 2. Materials and Methods

### 2.1. Study Design, Setting and Participants

The Study of Health in Pomerania (SHIP) is a population-based cohort study involving volunteers aged 20–79 from the Northeast German region of Mecklenburg-Western Pomerania. The goal of SHIP is to obtain longitudinal epidemiological insight into disease prevalence and incidence, common risk factors and lifestyle choices. SHIP was approved by the Ethics Committee of University Medicine Greifswald (IIIUV73/01, 12 December 2001) and conducted in accordance with the Declaration of Helsinki. Participants are selected through a structured random sampling process. Three independent cohorts (SHIP-START, SHIP-TREND and SHIP-NEXT) have been concurrently recruited.

This study is a secondary analysis of data from the third follow-up of SHIP-START (START-3), collected between 2014 and 2016, which included whole-body MRI assessments with a 1.5 T device (Magnetom Avanato, Siemens Healthcare, Erlangen, Germany). All of the 1717 volunteers participating in this follow-up were offered the opportunity to undergo whole-body MR imaging; of these, 870 agreed to the examination. The data use application for this project was approved by the Executive Board of the Community Medicine Research Network at University Medicine Greifswald (Data Use Application SHIP 2023/97/D).

In order to avoid susceptibility bias in the heterogenic cohort of 20–79-year-old participants, an additional sensitivity analysis restricting the sample to participants meeting more stringent selection criteria was conducted [age > 50 years and absence of inflammatory joint disease (*n* = 32, 3.8%), hypotension (*n* = 49, 5.7%), and osteoporosis (*n* = 58, 6.8%), *n* = 496]. The results, however, are comparable to those in the full data set without selection criteria, and the selection introduces instability to the model due to the reduced sample size. Therefore, we refrained from presenting those data and stuck to the whole cohort.

### 2.2. Variables

#### MRI-Based Measurement of Spondylophytes

**Figure 1:**
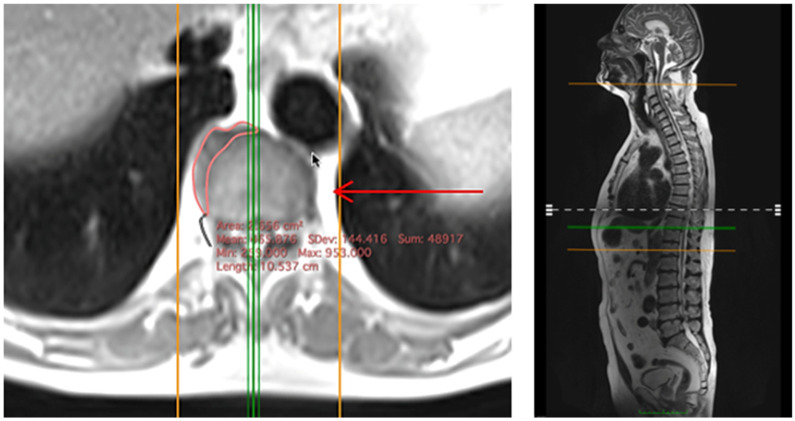
MRI-based measurement of thoracic spondylophytes. Spondylophyte size at T8–T11 was measured on axial T2-weighted MRI.

Right-sided spondylophytes at vertebral levels T8–T11 were assessed using T2-weighted MRI sequences: a sagittal sequence provided a high-contrast overview of the thoracolumbar spine with minimal motion artifacts, while an axial sequence allowed precise visualization of individual spondylophytes (sequence parameters: Repetition time = 3230 ms, Echo time = 34 ms, Flip angle = 180°, Voxel size = 1.6 mm × 1.6 mm × 3.0 mm, Duration = 2.43 min, Resolution = 512 pixels × 512 pixels, Pixel bandwidth = 170 Hz/pixel) (Figure 1).

Measurements were conducted in the DICOM-viewer Horos (Version: Horos 4.0.1, Manufacturer: Horos Project, Inc. (USA), Annapolis, MD, USA). The axial slice showing the largest spondylophyte extent was selected, and the spondylophyte’s boundary was traced using the closed polygon tool to determine its area.

Inter-reader variability was assessed by two readers independently evaluating 200 participants (800 vertebrae) for both presence and area of thoracic spondylophytes. Reader 1, a radiology resident with 2 years of experience, and Reader 2, a medical student following a standardized instruction in spinal anatomy and spondylophyte assessment, showed good to substantial agreement when rating the presence of spondylophytes (Cohen’s κ = 0.79), and good reliability when measuring the area of spondylophytes (intraclass correlation coefficient, ICC = 0.77). Both the resident and medical student were trained by a board-certified radiologist, who at random intervals also double-checked the measurements.

### 2.3. Hypertension and Covariates

The primary exposure variable arterial hypertension was defined as a pre-existing history of arterial hypertension or the previous prescription of relevant medication, assessed using a standardised questionnaire administered to all participants as part of SHIP, as well as hypertensive systolic blood pressure during all blood pressure assessments conducted in the course of the study. Hypertension was defined as a systolic blood pressure of ≥140 mmHg [21].

Fourteen additional covariates considered in the analysis were selected based on the literature and data availability, including age, gender, BMI, thyroid and cardiovascular diseases, diabetes, chronic pain, allergies, asthma, nicotine and alcohol use, physical activity, and physically demanding work.

### 2.4. Directed Acyclic Graph

**Figure 2:**
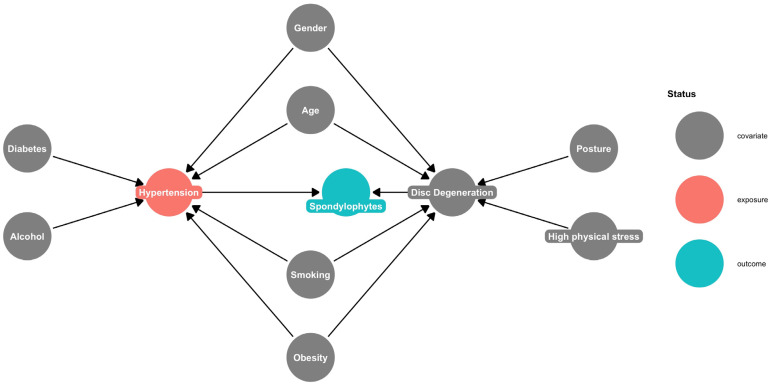
Directed acyclic graph illustrates hypothesized causal paths between hypertension (exposure, red), spondylophytes (outcome, blue), and covariates (grey) relevant for adjustment.

The directed acyclic graph (DAG) in Figure 2 illustrates the relationship between the response variable (spondylophytes, marked in blue) and the exposure variable (arterial hypertension, marked in red). Additionally, it shows the contributions of covariates to this relationship. Nodes represent variables, arrows depict the direction of influence and highlight potential causal paths.

Spondylophytes are directly influenced by hypertension and disc degeneration. Gender, age, smoking and obesity directly influence both hypertension and disc degeneration. Diabetes and alcohol consumption mainly impact hypertension, while posture and high physical stress impact only disc degeneration.

### 2.5. Statistical Analysis

Statistical analysis was conducted using R Version 4.4.2 (R Foundation for Statistical Computing, Vienna, Austria) and independently validated in SPSS Version 30 (IBM Corporation, Armonk, NY, USA).

Descriptive statistics were calculated for the entire study population using all available data. For regression and machine learning analyses, complete-case analysis was applied.

The directed acyclic graph (DAG) was created using the R package ggdag [22]. The minimal adjustment set was identified using ggdag::ggdag_adjustment_set, yielding age, sex, obesity (BMI ≥ 30), smoking and disc degeneration as sufficient covariates to block all non-causal paths between hypertension and spondylophyte formation [23,24]. These variables were therefore included as covariates in all multivariate regression models. However, disc degeneration could not be analysed due to missing information. From a causal inference perspective, this omission does not introduce bias into the estimation of the hypertension–spondylophyte association, as disc degeneration functions as a pure outcome predictor in the DAG—it affects spondylophyte formation but not hypertension. Excluding such variables does not open any backdoor paths between exposure and outcome, and therefore does not compromise the validity of the adjustment strategy, though it may slightly reduce precision [25,26,27]. All remaining variables listed in Table 1 were included for descriptive purposes only and were not entered into the regression models.

Multivariate regression models were used to assess associations between spondylophytes, hypertension, and relevant covariates. Logistic regression was used to model the binary outcome (presence vs. absence), while linear regression was applied to the continuous outcome of spondylophyte size at vertebrae T8–T11.

In addition to standard regression techniques, we employed machine learning approaches: First, linear discriminant analysis (LDA) was used as a classification method to identify linear combinations of features maximizing class separation, providing feature importance scores comparable to logistic regression. Second, random forest models were applied for both classification and regression, using an ensemble of decision trees with bootstrap aggregation; feature importance was assessed by the mean decrease in accuracy (classification) and mean increase in mean squared error upon variable permutation (regression). Third, a feedforward neural network with two hidden layers (16 and 8 nodes, ReLU activation function) was trained for regression to capture potential non-linear relationships between covariates and spondylophyte size. These methods were selected to provide complementary perspectives on feature importance from both linear and non-linear frameworks, allowing assessment of the robustness of the regression findings across methodologically distinct approaches. Model performance was assessed using R^2^, mean squared error, accuracy, sensitivity, and specificity [28,29].

This manuscript is compliant with the Strengthening of the Reporting of Observational Studies in Epidemiology (STROBE) statement [30].

## 3. Results

### 3.1. Participants

**Figure 3:**
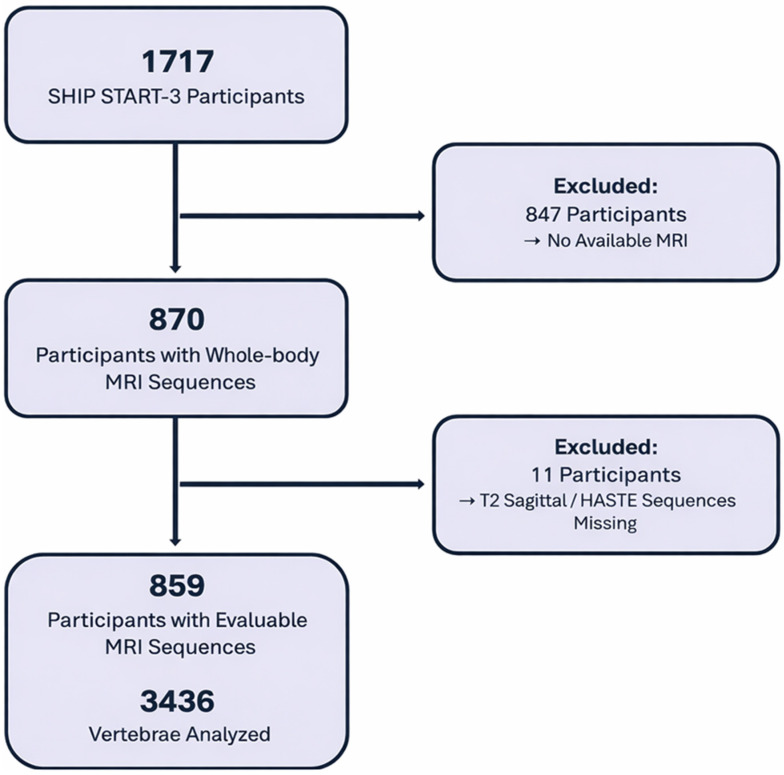
Flowchart of the study population.

As illustrated in Figure 3, 870 participants with whole-body MRI were included out of 1717 study participants (SHIP START-3). Of these, 11 participants had to be excluded due to the absence of suitable T2-weighted MRI sequences of the spine. The final study population consisted of 859 participants with 3436 vertebrae analysed.

Participants with available spondylophyte information analysed in this study (*n* = 859, 50%) were significantly younger, had a lower BMI and waist-to-hip ratio, were less often obese, and were more frequently highly physically active compared to participants without spondylophyte data (*n* = 858, 50%). They were less likely to engage in physically demanding work but more likely to perform computer-based work, defined as computer screen use for at least six months since the last SHIP examination. Furthermore, they exhibited lower rates of hypertension, a higher prevalence of diabetes, and a greater occurrence of degenerative spinal conditions such as herniated discs, sciatica, or lumbago (see Supplementary Table S1).

Table 1 summarizes the characteristics of the whole study sample, stratified for presence or absence of spondylophytes. Missing values are indicated in the last column. Participants analysed in this study were on average 58.8 (±12.3) years old, 467 (54.4%) were female, and 433 (50.5%) had hypertension with a median duration of 12.0 years since diagnosis (IQR 5.0–21.8). The presence of thoracic spondylophytes in any of the T8-T11 vertebrae was observed in 753 (87.7%) participants. Participants with spondylophytes showed a significantly higher prevalence and longer duration of hypertension (prevalence 54.9% vs. 19.8%, *p* < 0.001; median duration 12 years (IQR 5–22) vs. 5 years (IQR 2–10.5), *p* = 0.002, respectively). They were also older and exhibited a higher BMI, waist-to-hip ratio, and systolic and diastolic blood pressure, while hypotension was less common. Additionally, they more frequently had diabetes (specifically of type 2), obesity, elevated blood lipids, joint wear, and disc prolapse. In addition, the association between sex and presence of spondylophytes is significant (*p* < 0.001), with men being less likely to have no spondylophytes (19.8% vs. 80.2%) compared to women.

Missingness for the primary exposure variable (hypertension: 0.2%) and all covariates included in the regression models (age: 0%, sex: 0%, obesity: 0%, smoking: 0.1%) was negligible. Variables with higher rates of missing data, such as hypertension duration (53.2%) and physically demanding work (34.2%), were included for descriptive purposes only and were not entered into the regression models.

### 3.2. Thoracic Spondylophytes

**Figure 4:**
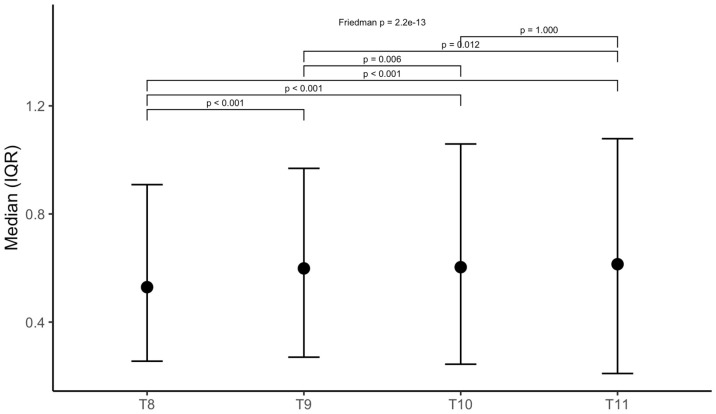
Spondylophyte sizes presented as median and quartiles for T8-T11; Friedman and pairwise Wilcoxon significance test.

In cases where participants exhibited spondylophytes, their sizes were significantly higher in those with hypertension compared to those without at vertebra T8 (median size 0.69 (IQR 0.39–1.05) vs. 0.62 (IQR 0.40–0.86), *p* = 0.039, see Table 2). This effect was not observed for vertebrae T9-T11. However, participants with hypertension exhibited spondylophytes significantly more often compared to participants without (95.2% vs. 80.0%, *p* < 0.001). Consequently, when the absence of spondylophytes was coded as size zero, significant size differences were observed across all vertebrae from T8 to T11. Spondylophyte size increased significantly from vertebrae T8 to T11 (see Figure 4).

#### Association Between Arterial Hypertension and Thoracic Spondylophytes

**Figure 5:**
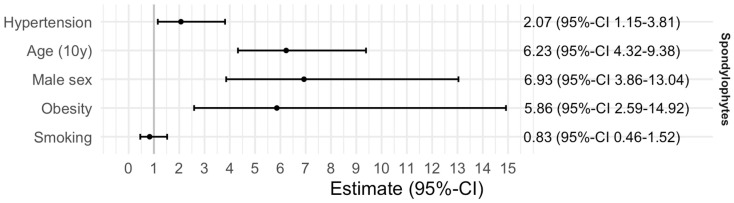
Association of hypertension with spondylophyte presence adjusted for covariates.

**Figure 6:**
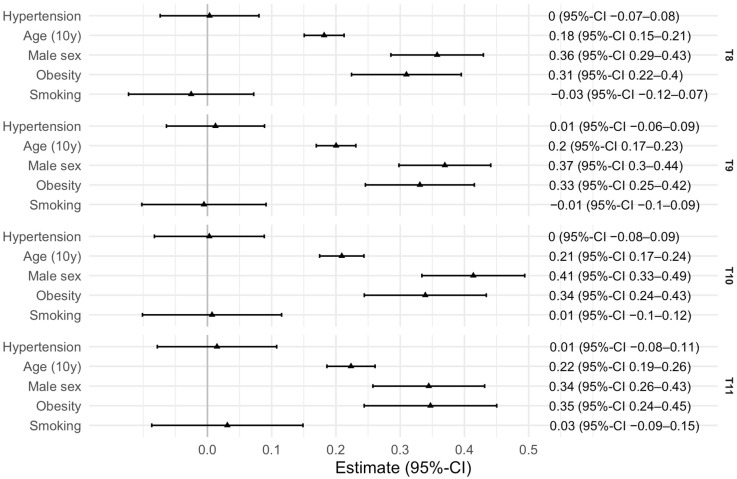
Association of hypertension with spondylophyte size at T8–T11, adjusted for covariates.

In Figure 5 and Figure 6, results from regression analyses are presented. Logistic regression for the binary outcome of spondylophyte presence yielded significant effects for hypertension (OR: 2.07, 95%-CI: 1.15–3.81), a 10-year increase in age (OR: 6.23, 95%-CI: 4.32–9.38), male sex (OR: 6.93, 95%-CI: 3.86–13.04), and obesity (OR: 5.86, 95%-CI: 2.59–14.92), which were associated with a higher likelihood of spondylophytes and larger spondylophyte sizes across T8–T11. The odds ratio for hypertension is 2.07 (95% CI: 1.15–3.81), suggesting a potentially increased risk.

In linear regression analysis, conducted to analyse the association between the size of spondylophytes and hypertension, and adjusted for the covariates, the effects of age, male sex and obesity could be found again. However, the inclusion of 1 within the confidence interval for hypertension indicates no statistical significance. Smoking does not appear to have a significant impact on either outcome. Simple linear regression models further confirmed that each additional decade of age was associated with an increase in spondylophyte size, with 0.18 (0.15–0.21) cm^2^ for T8, 0.20 (0.17–0.23) cm^2^ for T9, 0.21 (0.18–0.24) cm^2^ for T10 and 0.22 (0.19–0.26) cm^2^ for T11.

Multicollinearity was not present (variance inflation factors of 1.03, 1.12, 1.10, 1.06, and 1.01 for hypertension, age decades, sex, obesity, and smoking). The adjusted R^2^ values in the multiple regression models ranged from 0.283 to 0.363 in linear regression and was 0.505 in logistic regression.

### 3.3. Model Validation and Sensitivity Analysis

We conducted several sensitivity analyses to assess the stability of our results beyond the initially made assumptions and modelling choices.

### 3.4. Sensitivity Analyses

(1) Since clinical diagnoses often miss subclinical cases, we defined hypertension at the study visit—per the European Society of Cardiology’s Guidelines for the management of elevated blood pressure and hypertension 2024—as the average of three blood-pressure readings ≥ 140 mmHg [21]. We then created a composite hypertension variable (history of diagnosis or elevated visit readings), resulting in 507 (59.2%) patients classified as hypertensive. Both univariate and multivariate analyses yielded the same results. (2) Replacing the binary obesity variable with continuous BMI abolished the previously observed effect of hypertension. (3) When replacing the binary variable smoking with the number of cigarettes smoked per day, the effects remained unchanged. (4) The same is true when entering the variable regular physical activity into the model. (5) Statistical analyses were independently validated in SPSS, producing fully consistent results.

### 3.5. Model Validation Using Machine Learning Methods

We validated the regression results by applying multiple machine and statistical learning algorithms and comparing feature importance across covariates: For spondylophyte presence, LDA ranked hypertension as the fourth most important factor (score: 0.441), after sex (0.838), age (0.735), and obesity (0.617), and before smoking (0.008), aligning closely with logistic regression. In contrast, the random forest model ranked hypertension as the least important predictor, even assigning it a negative value in mean decrease in accuracy, suggesting that its removal could improve model performance. For spondylophyte size, random forest models showed consistent rankings across all four vertebrae. Feature importance was measured by the percentage increase in mean squared error (MSE) upon removal. Hypertension consistently ranked fourth (8.5–12.0%), after sex (24.5–28.7%), age (24.4–26.6%), and obesity (21.0–24.5%), and before smoking (3.3–8.8%). Only at vertebra 11 did age (26.5%) slightly outrank sex (24.5%). The feedforward neural network (2 hidden layers: 16 and 8 nodes, ReLU activation) yielded similar results. Permutation feature importance ranked age highest (MSE increase: 0.255), followed by sex (0.194), obesity (0.116), hypertension (0.056), and smoking (0.035). Overall, hypertension did not emerge as a major predictor of spondylophyte size, but both LDA and logistic regression consistently identified it as relevant for predicting presence, highlighting its potential role in classification tasks. In contrast, results for spondylophyte size were highly consistent across all regression models, with age, sex, and obesity repeatedly ranked as the most influential factors.

## 4. Discussion

This study investigated the association between arterial hypertension and thoracic spondylophyte formation in a population-based cohort using whole-body MRI of 859 adults. We observed a high prevalence of thoracic spondylophytes (87.7%) between vertebrae T8 and T11. Hypertension was significantly associated with their presence after adjusting for age, sex, obesity, and smoking (OR = 2.07, 95% CI: 1.15–3.81). No consistent association was found between hypertension and spondylophyte size. Spondylophyte size increased progressively from T8 to T11 and was strongly associated with age, male sex, and obesity. Participants with spondylophytes were older, had higher BMI and blood pressure, and more frequently showed metabolic and degenerative comorbidities. These findings were robust across sensitivity analyses and were further supported by machine learning models.

### 4.1. Limitations

Before interpreting our findings, several limitations must be acknowledged. First, selection bias is possible, as the analysis was limited to participants with whole-body MRI data, forming a non-random subset of the SHIP-START-3 cohort. This may reduce the generalizability of the results. Second, the primary hypertension definition relied on self-reported diagnosis and antihypertensive medication use, which is susceptible to information bias and misclassification. Participants unaware of their hypertension would be incorrectly classified as normotensive, while treated patients with well-controlled blood pressure would be classified as hypertensive regardless of their current values. To address this, a pre-specified sensitivity analysis was conducted using the 2024 ESC definition based on the mean of three objective blood pressure measurements (≥140 mmHg), resulting in a composite variable classifying 59.2% of participants as hypertensive compared to 50.5% under the primary definition. Key findings remained stable across both definitions, suggesting that misclassification bias had limited impact on the observed associations. Third, the broad age range of the cohort (20–79 years) introduces potential susceptibility bias, as spondylophyte prevalence increases substantially with age, and the age distribution of participants may therefore influence the observed associations. Although age was included as a continuous covariate in all multivariate models, residual age-related confounding cannot be fully excluded. To further address this concern, an additional sensitivity analysis restricting the sample to participants meeting more stringent age criteria is reported in the Supplementary Material. Regarding baseline spinal status, whole-body MRI was introduced into the SHIP cohort only at the START-3 wave, meaning no prior imaging data are available for this population. The absence of baseline spinal imaging therefore precludes assessment of the temporal sequence between hypertension onset and spondylophyte development, and the possibility that spondylophytes preceded hypertension cannot be excluded. Fourth, model stability varied with covariate specification: the association between hypertension and spondylophytes was significant when obesity was treated as a binary variable, but not when BMI was included continuously. However, results remained consistent across alternative specifications for smoking and physical activity, and machine learning models confirmed the regression findings, strengthening confidence in the observed associations. Fifth, residual confounding remains possible. Unmeasured factors or imprecisely captured variables, such as BMI as a surrogate for body composition, may affect both hypertension and spondylophyte formation. Sixth, this study establishes a general correlation between back pain and spinal spondylophyte status. However, it cannot demonstrate a precise symptom correlation between reported back pain and measured spondylophytes at the T8–11 level in this specific cohort. The section of the spine under investigation was chosen because of the most frequent location of spondylophytes in the thoracic spine being at the level of T8–11 [7,31] and its proximity to the aorta [8]. Finally, the cross-sectional nature of the underlying cohort data precludes causal inference, and the lack of detailed diagnostic timing for hypertension further limits conclusions about directionality. Future longitudinal studies should incorporate repeated imaging, standardized clinical assessments, and biomechanical parameters to better understand causality.

### 4.2. Clinical and Epidemiological Relevance

The association between arterial hypertension and thoracic spondylophytes observed in this study adds a vascular dimension to our understanding of spinal degeneration. Hypertension is highly prevalent in patients with back pain in Germany [32], and this overlap underscores the need to explore cardiovascular contributions to spinal bone remodelling. While spondylophytes are commonly seen in spinal imaging, especially in older adults, their clinical significance remains unclear, as they are frequently incidental and do not consistently correlate with symptoms [33,34]. Unlike syndesmophytes, which are strongly associated with inflammatory conditions such as ankylosing spondylitis [35], spondylophytes may not be direct markers of pain or functional impairment. This is supported by imaging studies of lumbar degeneration that have failed to show a robust link between radiological findings and clinical symptoms [34]. However, their higher prevalence in hypertensive individuals raises the possibility of vascular contributions to spinal ageing, which may influence the broader pathophysiology of back pain. Understanding this relationship may support risk assessment in patients with vascular comorbidities, may support further investigation into the interplay between hemodynamics and skeletal adaptation, and could have implications for prevention and risk stratification.

### 4.3. Established Mechanical View

Intervertebral disc degeneration is widely considered a prerequisite for spondylophyte development. However, it remains unclear to what extent biomechanical or metabolic risk factors influencing disc degeneration also apply to spondylophyte formation [36]. While most existing literature has focused on disc degeneration or anterior longitudinal ligament ossification, particularly in the context of diffuse idiopathic skeletal hyperostosis (DISH), systemic vascular contributions have received comparatively little attention. Besides vascular mechanisms, mechanical loading, aging-related degeneration, genetic predisposition, and metabolic factors (e.g., obesity and dyslipidemia) are also recognized contributors to spondylophyte development. These factors may interact with hypertension, suggesting that the observed association is likely multifactorial rather than causal in a unidirectional sense.

### 4.4. Systemic Vascular Contributions to Vertebral Cortical Remodelling

The observed association between hypertension and spondylophyte presence, but not size, may reflect a vascular influence on early rather than advanced stages of bony outgrowth. Elevated blood pressure, through increased arterial pulsatility, could induce localized microloading at the vertebral margins. The literature hypothesizes that the pulsatile movement of the aorta may create a local biomechanical environment unfavorable for ossification, but the exact cellular or molecular pathways remain undetermined [11]. Similar to rib notching in aortic coarctation, where mechanical stress causes cortical remodelling [37], chronic hemodynamic strain may influence osteoproliferation through adaptive or stress-related mechanisms in susceptible individuals. The potential role of vascular factors in spinal bone remodelling has received limited attention compared to mechanical and inflammatory pathways. However, several studies point to plausible molecular mechanisms through which vascular contributions could affect osteogenesis.

### 4.5. Vascular Influences in Bone Formation

Beyond mechanical and biomechanical influences, molecular mediators originating from vascular pathways have also been linked to pathological bone formation. Vascular factors contribute to pathological bone formation in the spine through angiogenesis-dependent mechanisms, endothelial-to-mesenchymal transition (EndMT), and pro-osteogenic signaling pathways. The specific vascular mechanisms vary across different spinal ossification disorders. VEGF-mediated angiogenesis is essential for spinal osteophyte and syndesmophyte formation. In osteoarthritis, VEGF contributes to osteophyte formation through multiple mechanisms including cartilage degeneration, subchondral bone changes, and direct osteogenic effects [38]. Chondrogenic progenitor cells in osteoarthritic joints promote VEGF expression through stromal-derived factor-1α (SDF-1α), creating a paracrine loop that stimulates vascular invasion and osteophyte formation [39]. Increased skeletal VEGF enhances β-catenin activity through VEGFR2 and PI3K-mediated pathways, resulting in excessively ossified bones [40]. Inflammation-driven vascular changes promote pathological spinal ossification. In axial spondyloarthritis, inflammatory granulation tissue at the junction of the annulus fibrosus and vertebral bone leads to syndesmophyte formation through osteoblast proliferation. IL-23 and IL-17 stimulate mesenchymal stem cells to differentiate into osteoblasts, leading to pathologic new bone formation [41,42]. Elevated levels of vascular endothelial growth factor (VEGF), for example, have been linked to spinal progression in axial spondylarthritis, although findings remain inconsistent: while some studies found no significant association with syndesmophyte formation [43,44], others reported that higher baseline VEGF levels predicted radiographic progression [45]. Similarly, platelet-derived growth factor B (PDGFB) and hepatocyte growth factor (HGF) have been implicated in syndesmophyte development in ankylosing spondylitis [46,47]. Although these data are derived from inflammatory contexts, they highlight the capacity of vascular-derived mediators to influence new bone formation. Endothelial cells directly contribute osteoprogenitor cells to spinal heterotopic ossification through EndMT. Vascular endothelial cells differentiate into skeletal cells through a mesenchymal stem cell intermediate, with local inflammatory signals and tissue microenvironment changes mediating differentiation into chondrocytes and osteoblasts [48]. All these mechanisms raise the further question of whether similar mechanisms are also effective in non-inflammatory diseases such as hypertension. Observational data from patients with DISH demonstrate an increased burden of coronary atherosclerosis, a condition characterized by excessive spinal bone formation, reinforcing the concept of a systemic vascular–skeletal interaction [49]. Finally, while intervertebral disc degeneration is traditionally viewed as a prerequisite for spondylophyte formation, it remains unclear whether the same biomechanical and metabolic factors apply equally to spondylophytes, or whether vascular influences act independently [36].

### 4.6. Generalizability

Our imaging-based findings provide structural evidence supporting the hypothesis that vascular factors, particularly hypertension, may contribute to spinal bone remodelling and aging. These results expand current biomechanical models by introducing vascular influences as a potential systemic modifier of spinal structure. However, the nature of this secondary analysis limits causal inference, and the exploratory nature of the study requires cautious interpretation. Given the heterogeneity in the existing literature, our findings should be considered hypothesis-generating and warrant confirmation in longitudinal and diverse population-based settings. Nevertheless, this study is the first of its kind that, based on the evaluation of MRI images and personal information from a large sample of a representative population study, allows conclusions to be drawn about the influence of hypertension and other selected variables on spondylophyte growth.

## 5. Conclusions

In this population-based imaging cohort, arterial hypertension was modestly but independently associated with the presence of thoracic spondylophytes after adjustment for major demographic and metabolic confounders. These findings are consistent with, but do not establish, the hypothesis that vascular factors may contribute to processes involved in spinal bone remodelling, particularly in non-inflammatory contexts. Within the framework of existing mechanical and molecular models, our data raise the possibility that hypertension could act as a systemic correlate of spinal aging rather than a direct driver. As causal inference cannot be drawn from this cross-sectional secondary analysis, future longitudinal and mechanistic studies are required to determine whether thoracic spondylophytes represent epiphenomena of shared aging-related processes, structural markers of vascular burden, or entities with independent relevance for musculoskeletal pathology. If it could be proven that spondylophyte growth is promoted by hypertension, further studies could investigate to what extent improved blood pressure management could reduce the prevalence of spondylophytes and thus reduce effects such as back pain.

## Figures and Tables

**Table 1:** Study participant characteristics.

	Total	Spondylophytes		*p*-Value	Missing
		No	Yes		
	859	106 (12.3%)	753 (87.7%)		n (%)
Age (yrs), mean (sd)	58.76 (12.2)	45.13 (8.1)	60.68 (11.5)	<0.001	0 (0)
Sex, *n* (%)					
Male	392 (45.6)	21 (19.8)	371 (49.3)	<0.001	0 (0)
Female	467 (54.4)	85 (80.2)	382 (50.7)		
BMI, mean (sd)	27.55 (4.55)	24.30 (3.28)	28.00 (4.52)	<0.001	0 (0)
Obesity, *n* (%)	218 (25.4)	8 (7.5)	210 (27.9)	<0.001	0 (0)
Waist-to-hip ratio, mean (sd)	0.92 (0.09)	0.85 (0.07)	0.93 (0.08)	<0.001	0 (0)
Physical exercise winter, *n* (%)					
Infrequent/None	344 (40.1)	43 (40.6)	301 (40.0)	1.000	1 (0.1)
Regular (≥1 h)	514 (59.9)	63 (59.4)	451 (60.0)		
Physical exercise summer, *n* (%)					
Infrequent/None	234 (27.3)	24 (22.6)	210 (27.9)	0.304	1 (0.1)
Regular (≥1 h)	624 (72.7)	82 (77.4)	542 (72.1)		
Regular physical activity, *n* (%)	502 (58.5)	62 (58.5)	440 (58.5)	1.000	1 (0.1)
Physically demanding work, *n* (%)	145 (25.7)	24 (24.5)	121 (25.9)	0.869	294 (34.2)
Duration in years, median (IQR)	5.0 (3.0, 15.0)	5.0 (2.8, 10.3)	5.0 (3.0, 16.0)	0.507	714 (83.1) *
Monitor work over at least 1/2 year	383 (67.8)	72 (73.5)	311 (66.6)	0.228	294 (34.2)
since last SHIP examination, *n* (%)					
Duration in years, median (IQR)	5.0 (4.0, 10.0)	5.00 (4.0, 11.0)	5.0 (4.0, 10.0)	0.408	476 (55.4) *
Current smoking, *n* (%)	146 (17.0)	27 (25.5)	119 (15.8)	0.019	1 (0.1)
Smoking starting age, median (IQR)	17.0 (16.0, 19.0)	16.0 (15.5, 18.0)	18.0 (16.0, 19.8)	0.172	714 (83.1) *
Number of cigarettes, median (IQR)	11.0 (8.0, 15.0)	12.0 (7.0, 15.0)	10.0 (8.0, 15.0)	0.926	13 (1.5)
Alcohol consumption (ever), *n* (%)	852 (99.3)	106 (100.0)	746 (99.2)	0.764	1 (0.1)
Amount of alcohol **, median (IQR)	0.0 (0.0, 1.0)	0.0 (0.0, 0.0)	0.0 (0.0, 1.0)	0.079	104 (12.1)
Systolic blood pressure, mean (sd)	132.59 (15.85)	122.13 (13.94)	134.06 (15.56)	<0.001	0 (0)
Diastolic blood pressure, mean (sd)	78.41 (9.33)	76.09 (8.97)	78.74 (9.34)	0.006	0 (0)
Heart rate, mean (sd)	66.86 (10.17)	67.84 (9.98)	66.72 (10.20)	0.290	0 (0)
Hypertension diagnosed, *n* (%)	433 (50.5)	21 (19.8)	412 (54.9)	<0.001	2 (0.2)
Duration, median (IQR)	12.0 (5.0, 21.8)	5.00 (2.0, 10.5)	12.0 (5.0, 22.0)	0.002	457 (53.2) *
Hypertension combined, *n* (%) ^#^	507 (59.2)	26 (24.5)	481 (64.0)	<0.001	2 (0.2)
Diabetes, *n* (%)	74 (8.6)	2 (1.9)	72 (9.6)	0.014	1 (0.1)
Diabetes type, n (%)					
Type 1	4 (5.5)	1 (50.0)	3 (4.2)	<0.001	786 (91.5) *
Type 2	67 (91.8)	0 (0.0)	67 (94.4)		
Gestational diabetes	2 (2.7)	1 (50.0)	1 (1.4)		
Duration, median (IQR)	9.0 (4.0, 15.0)	11.0 (9.5, 12.5)	9.0 (3.8, 15.5)	0.764	789 (91.9) *
Asthma, *n* (%)	49 (5.7)	8 (7.5)	41 (5.5)	0.518	1 (0.12)
Asthma attack ***, *n* (%)	10 (1.2)	0 (0.0)	10 (1.3)	0.477	1 (0.12)
Duration, median (IQR)	24.0 (13.0, 37.5)	25.0 (24.0, 37.5)	22.0 (11.8, 36.8)	0.410	816 (95.0) *
Cancer, *n* (%)	48 (5.6)	5 (4.7)	43 (5.7)	0.848	0 (0)
Thyroid disease, *n* (%)	221 (25.8)	24 (22.9)	197 (26.2)	0.539	2 (0.23)
Duration, median (IQR)	13.5 (6.0, 18.8)	11.0 (5.0, 18.0)	14.0 (6.0, 19.0)	0.878	669 (77.9) *
Hypotension, *n* (%)	49 (5.7)	12 (11.3)	37 (4.9)	0.015	0 (0)
Joint wear, *n* (%)	224 (26.2)	11 (10.5)	213 (28.4)	<0.001	5 (0.6)
Disc prolapse, *n* (%)	202 (23.6)	15 (14.4)	187 (24.9)	0.026	3 (0.4)
Inflammatory joint disease, *n* (%)	32 (3.8)	3 (2.9)	29 (3.9)	0.814	12 (1.4)
Osteoporosis, *n* (%)	58 (6.8)	2 (1.9)	56 (7.5)	0.057	8 (0.9)
Elevated blood lipids, *n* (%)	205 (24.3)	6 (5.7)	199 (26.9)	<0.001	15 (1.8)
Pain ****, *n* (%)	382 (44.5)	51 (48.1)	331 (44.0)	0.483	0 (0)
Back pain *****, *n* (%)	480 (55.9)	62 (58.5)	418 (55.5)	0.636	0 (0)
Back pain intensity, median (IQR)	3.0 (1.0, 6.0)	3.0 (0.5, 5.0)	4.0 (1.0, 6.0)	0.037	477 (55.5)

Values are presented as mean (SD) or median (IQR, i.e., 1st and 3rd quartiles) for continuous variables and *n* (%) for categorical variables. Percentages for the ‘No Spondylophytes’ and ‘Yes Spondylophytes’ columns are calculated within each subgroup. * Missing values include “no” answers in the respective variable. ** number of days with 5+ glasses in last month, *** within past 12 months, **** within the last week, ***** within the last 3 months, ^#^ hypertension defined as history of hypertension or mean of the three study-related measurements 140 mmHg.

**Table 2:** Spondylophyte sizes for T8–11 for all participants and stratified for hypertension.

	Total	Hypertension		*p*-Value	Missing
		No	Yes		
Patients *n* (%)	859	424 (49.4)	433 (50.4)		2 (0.2)
Spondylophytes, *n* (%)	753 (87.7)	339 (80.0)	412 (95.2)	<0.001	0 (0)
Spondylophyte size, median (IQR)					
T8 *	0.65 (0.39, 0.97)	0.62 (0.40, 0.86)	0.69 (0.39, 1.05)	0.039	137 (16.0)
T9 *	0.70 (0.41, 1.08)	0.68 (0.40, 0.95)	0.73 (0.42, 1.17)	0.060	133 (15.5)
T10 *	0.76 (0.43, 1.18)	0.77 (0.40, 1.10)	0.76 (0.44, 1.30)	0.124	154 (17.9)
T11 *	0.79 (0.46, 1.25)	0.79 (0.43, 1.15)	0.79 (0.48, 1.40)	0.290	185 (21.5)
T8 **	0.53 (0.26, 0.91)	0.47 (0.17, 0.80)	0.63 (0.30, 1.01)	<0.001	0 (0)
T9 **	0.60 (0.27, 0.97)	0.54 (0.18, 0.86)	0.66 (0.36, 1.14)	<0.001	0 (0)
T10 **	0.60 (0.24, 1.06)	0.51 (0.03, 0.94)	0.69 (0.33, 1.16)	<0.001	0 (0)
T11 **	0.61 (0.21, 1.08)	0.50 (0.00, 0.99)	0.69 (0.31, 1.21)	<0.001	0 (0)

* Calculated for participants with spondylophytes; ** calculated for all participants—in case of absence of spondylophytes, the size is zero.

## Data Availability

The data presented in this study are available on reasonable request from the corresponding author due to legal reasons.

## Supplementary Materials

The following supporting information can be downloaded at: https://www.mdpi.com/article/10.3390/healthcare14081024/s1, Table S1: Participant’s characteristics—stratified for analysis inclusion and exclusion.
