# Supplementary material for: Association of Arterial Hypertension with Thoracic Spondylophyte Formation: A Secondary Analysis of Cross-Sectional MRI Data from the SHIP Cohort
Source: Healthcare (Basel). 2026 Apr 13;14(8):1024. doi: 10.3390/healthcare14081024 (PMC13115812; doi:10.3390/healthcare14081024)
Supplement: Supplementary file 1 [file healthcare-14-01024-s001.zip › Suppl. Table.pdf]

Supplementary Table S1: Participant's characteristics - stratified for analysis inclusion and exclusion.

	Total	Study Cohort considered in analysis?		*p*-value	Missing
		No	Yes		
	1717	858 (50%)	859 (50%)		n (%)
Age (yrs), mean (sd)	60.11 (12.79)	61.46 (13.19)	58.76 (12.23)	<0.001	0 (0)
Sex, n (%)				0.647	0 (0)
Male	794 (46.2)	402 (46.9)	392 (45.6)		
Female	923 (53.8)	456 (53.1)	467 (54.4)		
BMI, mean (sd)	28.15 (5.02)	28.75 (5.38)	27.55 (4.55)	<0.001	5 (0.29)
Waist to hip ratio, mean (sd)	0.93 (0.09)	0.94 (0.09)	0.92 (0.09)	0.001	6 (0.35)
Obesity, n (%)	528 (30.8)	310 (36.3)	218 (25.4)	<0.001	5 (0.29)
Physical exercise winter, n (%)				0.001	20 (1.16)
Infrequent/None	748 (44.1)	404 (48.2)	344 (40.1)		
Regular (>= 1hour)	949 (55.9)	435 (51.8)	514 (59.9)		
Physical exercise summer, n (%)				0.001	19 (1.11)
Infrequent/None	528 (31.1)	294 (35.0)	234 (27.3)		
Regular (>= 1hour)	1170 (68.9)	546 (65.0)	624 (72.7)		
Regular physical activity, n (%)	920 (54.2)	418 (49.8)	502 (58.5)	<0.001	20 (1.16)
Physically demanding work, n (%)	304 (29.3)	159 (33.6)	145 (25.7)	0.006	679 (39.55)
- Duration in years, median (IQR)	5.0 (3.0, 13.3)	5.0 (3.0, 10.0)	5.0 (3.0, 15.0)	0.586	1413 (82.29)
Monitor work over at least 1/2 year since last SHIP examination, n (%)	654 (63.0)	271 (57.3)	383 (67.8)	0.001	679 (39.55)
- Duration in years, median (IQR)	5.0 (4.0, 11.0)	5.0 (4.0, 12.0)	5.0 (4.0, 10.0)	0.247	1064 (61.97)
Current smoking, n (%)	314 (18.5)	168 (20.0)	146 (17.0)	0.134	17 (0.99)

Smoking starting age, median (IQR)	17.0 (16.0, 19.0)	17.0 (15.0, 19.0)	17.0 (16.0, 19.0)	0.139	1406 (81.89)
Number of cigarettes, median (IQR)	12.0 (8.0, 15.3)	12.5 (8.0, 20.0)	11.0 (8.0, 15.0)	0.073	1433 (83.46)
Alcohol consumption (ever), n (%)	1683 (98.9)	831 (98.6)	852 (99.3)	0.222	16 (0.93)
Amount of alcohol**, median (IQR)	0.0 (0.0, 1.0)	0.0 (0.0, 0.0)	0.0 (0.0, 1.0)	0.150	267 (15.55)
Systolic blood pressure, mean (sd)	132.70 (16.70)	132.81 (17.51)	132.59 (15.85)	0.788	2 (0.12)
Diastolic blood pressure, mean (sd)	77.92 (9.97)	77.42 (10.56)	78.41 (9.33)	0.038	2 (0.12)
Heart rate, mean (sd)	67.47 (10.81)	68.09 (11.38)	66.86 (10.17)	0.018	2 (0.12)
Hypertension diagnosed, n (%)	937 (54.8)	504 (59.1)	433 (50.5)	<0.001	7 (0.41)
- Duration, median (IQR)	12.0 (5.0, 22.0)	12.0 (5.0, 21.8)	12.0 (5.0, 21.8)	0.584	849 (49.45)
Hypertension combined, n (%)[#]	1077 (63.1)	570 (67.0)	507 (59.2)	0.001	9 (0.52)
Diabetes, n (%)	203 (11.9)	129 (15.1)	74 (8.6)	<0.001	6 (0.35)
Diabetes type, n (%)				0.188	1521 (88.58)
Type 1	13 (6.6)	9 (7.3)	4 (5.5)		
Type 2	179 (91.3)	112 (91.1)	67 (91.8)		
Gestational diabetes	2 (1.0)	0 (0.0)	2 (2.7)		
?	2 (1.0)	2 (1.6)	0 (0.0)		
- Duration, median (IQR)	10.0 (4.0, 20.0)	11.0 (4.0, 20.3)	9.0 (4.0, 15.0)	0.154	1519 (88.47)
Asthma, n (%)	85 (5.0)	36 (4.2)	49 (5.7)	0.199	10 (0.58)
Asthma attack***, n (%)	21 (1.2)	11 (1.3)	10 (1.2)	0.987	7 (0.41)
- Duration, median (IQR)	21.0 (12.0, 36.0)	16.0 (7.8, 32.0)	24.0 (13.0, 37.5)	0.128	1642 (95.63)
Cancer, n (%)	93 (5.4)	45 (5.3)	48 (5.6)	0.877	9 (0.52)
Thyroid disease, n (%)	458 (26.8)	237 (27.9)	221 (25.8)	0.357	10 (0.58)
- Duration, median (IQR)	14.0 (6.0, 20.0)	15.0 (7.0, 23.3)	13.5 (6.0, 18.8)	0.200	1339 (77.98)
Hypotension, n (%)	96 (5.6)	47 (5.5)	49 (5.7)	0.959	8 (0.47)

Joint wear, n (%)	478 (28.2)	254 (30.1)	224 (26.2)	0.086	19 (1.11)
Disc prolapse, n (%)	447 (26.2)	245 (28.8)	202 (23.6)	0.016	11 (0.64)
Inflammatory joint disease, n (%)	77 (4.6)	45 (5.4)	32 (3.8)	0.145	34 (1.98)
Osteoporosis, n (%)	124 (7.4)	66 (7.9)	58 (6.8)	0.441	32 (1.86)
